# Air Pollution’s Impact on Cardiac Remodeling in an Experimental Model of Chagas Cardiomyopathy

**DOI:** 10.3389/fcimb.2022.830761

**Published:** 2022-07-19

**Authors:** Keila Cardoso Barbosa Fonseca, Fernanda Gallinaro Pessoa, Orlando do Nascimento Ribeiro, Viviane Tiemi Hotta, Barbara Maria Ianni, Fabio Fernandes, Dolores Helena Rodriguez Ferreira Rivero, Paulo Hilário Nascimento Saldiva, Charles Mady, Felix José Alvarez Ramires

**Affiliations:** ^1^ Instituto do Coração (InCor), Hospital das Clínicas da Faculdade de Medicina da Universidade de São Paulo (HCFMUSP), São Paulo, Brazil; ^2^ Department of Pathology, Experimental Air Pollution Laboratory, Laboratório de Investigação Médica 05 (LIM 05) - School of Medicine, University of São Paulo, São Paulo, Brazil

**Keywords:** Chagas disease, air pollution, myocardial fibrosis, cardiomyopathy, remodeling

## Abstract

**Background:**

Chagas disease is characterized by intense myocardial fibrosis stimulated by the exacerbated production of inflammatory cytokines, oxidative stress, and apoptosis. Air pollution is a serious public health problem and also follows this same path. Therefore, air pollution might amplify the inflammatory response of Chagas disease and increase myocardial fibrosis.

**Methods:**

We studied groups of Trypanosoma cruzi infected Sirius hamsters (Chagas=CH and Chagas exposed to pollution=CH+P) and 2 control groups (control healthy animals=CT and control exposed to pollution=CT+P). We evaluated acute phase (60 days post infection) and chronic phase (10 months). Echocardiograms were performed to assess left ventricular systolic and diastolic diameter, in addition to ejection fraction. Interstitial collagen was measured by morphometry in picrosirius red staining tissue. The evaluation of inflammation was performed by gene and protein expression of cytokines IL10, IFN-γ, and TNF; oxidative stress was quantified by gene expression of NOX1, MnSOD, and iNOS and by analysis of reactive oxygen species; and apoptosis was performed by gene expression of BCL2 and Capsase3, in addition to TUNEL analysis.

**Results:**

Chagas groups had increased collagen deposition mainly in the acute phase, but air pollution did not increase this deposition. Also, Chagas groups had lower ejection fraction in the acute phase (p = 0.002) and again air pollution did not worsen ventricular function or dilation. The analysis of the inflammation and oxidative stress pathways were also not amplified by air pollution. Apoptosis analysis showed increased expression of BCL2 and Caspase3 genes in chagasic groups in the acute phase, with a marginal p of 0.054 in BCL2 expression among infected groups, and TUNEL technique showed amplified of apoptotic cells by pollution among infected groups.

**Conclusions:**

A possible modulation of the apoptotic pathway was observed, inferring interference from air pollution in this pathway. However, it was not enough to promote a greater collagen deposition, or worsening ventricular function or dilation caused by air pollution in this model of Chagas cardiomyopathy.

## Introduction

Chagas disease is an infection of parasitic origin caused by a protozoan called Trypanosoma cruzi (T. cruzi). It was described by Carlos Ribeiro Justiniano Chagas to the scientific community in April 1909 (Chagas, 1909). According to the World Health Organization, it is one of the 20 most neglected tropical diseases in the world (Álvarez-Hernández et al., 2020). It is a serious health problem in many countries in Latin America, and it has spread to North America, Europe, and Asia. It is estimated that 8 million people are infected worldwide, with 6 to 7 million in 21 countries in Latin America (Sangenito et al., 2020). In recent years, oral transmission of the disease has been a major contributor to the increased number of acute new cases.

Chagas disease is considered a collection of several pathophysiology mechanisms that involves many interconnected pathways, including the inflammatory process coupled with oxidative stress causing programmed cell death (apoptosis). These mechanisms are important contributors to cardiac dysfunction and pathogenesis during chronic infection from Chagas disease (Brazão et al., 2015). This cardiomyocyte loss may cause collagen replacement and make Chagas cardiomyopathy the most myocardial fibrotic among all cardiomyopathies. Furthermore, such fibrosis may be responsible for histopathological pro-arrhythmogenic changes and sudden death in this population (Rodriguez, 2020; Barizon et al., 2020).

On the other hand, air pollution is a public health problem affecting large, urbanized centers. According to WHO, more than 7 million deaths per year are caused by the effects of urban pollution (WHO, 2020). In metropolitan areas, air pollution has been a threat to the quality of life and health of its inhabitants.

Among these pollutants is particulate matter (PM) that is made up of dust, fumes, and various solid and liquid materials that remain in the atmosphere because of their small size. PM is classified by size and consequently by its potential to cause disease. Currently, some studies have associated air pollution with the occurrence of cardiovascular diseases through different mechanisms, such as inflammation, oxidative stress, and apoptosis (Li et al., 2017; Rao et al., 2018; Miller and Newby, 2020), the same as those stimulated by T. cruzi infection. A study developed by our group associating atmospheric pollution with cardiac remodeling has shown increased myocardial fibrosis (de Oliveira-Fonoff et al., 2017).

Because Chagas disease promotes the activation of a complex chain of events with intense inflammation, oxidative stress, and apoptosis resulting in intense myocardial fibrosis, and air pollution being an important factor in stimulating these same cascades of events, we hypothesized that air pollution would amplify these responses and increase the myocardial fibrosis in an animal model of Chagas disease. We, therefore, evaluated the role of air pollution in ventricular remodeling in experimental Chagas disease.

## Materials and Methods

Animal model: We studied 100 female Sirius hamsters that were 5 to 8 weeks old. These animals were divided into 4 groups: control healthy animals not exposed to air pollution (CT, 25 animals), control exposed to air pollution (CT+P, 25 animals), chagasic not exposed to pollution (CH, 25 animals), and chagasic exposed to pollution (CH+P, 25 animals). The chagasic hamsters were infected intraperitoneally with 105 trypomastigote forms of T. cruzi Y strain diluted in saline. At 60 days into the protocol, defined as the acute phase, 10 animals from control groups were sacrificed to serve as controls for this phase. In addition, an analysis of the chronic phase (animals surviving at 10 months) was performed. The euthanasia was induced with the animals under ketamine and xylazine anesthesia. The protocol was performed in accordance with the recommendations of the Brazilian Directive for the Care and Use of Animals for Scientific and Didactic Purposes (DBCA - CONCEA) (CONCEA, 2016) and the Guide for the Care and Use of Laboratory Animals (National Research Council) (National Research Council, 2011). The study protocol was reviewed and approved by the ethics committee (CEUA: 151/15) of the Heart Institute and the University of São Paulo Medical School.

Exposure protocol: The exposure was performed as previously described (de Brito et al., 2018; Moreira et al., 2020). The animals were exposed through the stationary electric diesel exhaust generator (DE) (BD-2500 CFE; China). The type of fuel used was the metropolitan diesel oil (10 ppm of sulfur plus 5% soy biodiesel). The gases and particles from burning diesel fuel were stored in a BAG and subsequently, atmospheric air was captured, which was directed to the HEPA air filtration system and was responsible for diluting the air from generator exhaust. Then, gases and particles from the BAG were directed to the inhalation chamber, where the hamsters were accommodated. During exposure to diesel, the concentration of 600 μg/m3 of PM_2.5_ was controlled by the pressure regulator of the pneumatic gas pump. The hamsters were exposed for 2 hours twice a week. The concentrations of PM_2.5_ (DataRam4™, Thermo Fisher Scientific Inc., Franklin, MA) of nitric oxide (NO), nitrogen dioxide (NO2), temperature, and relative humidity (Thermo Fisher Scientific Inc., Franklin, MA) were monitored in real time.

Collagen morphometry: Serial paraffin sections (5 µm) from the formalin-fixed heart were stained with fibrillar collagen specific picrosirius red. Stained coronal sections from both ventricles were then viewed by light microscopy to identify sites of fibrosis, including those in the interstitial space. Interstitial collagen volume fraction (ICVF) of both ventricles were determined by videomorphometry using Leica QWIN image processing and analysis software (Leica Microsystems, Cambridge, United Kingdom) (Fernandes et al., 2012). The blinded examiner took all measurements.

Echocardiography: Transthoracic M-mode, two-dimensional, and pulsed Doppler echocardiography was performed using a GE Vivid E9 ultrasound system device with a 4 to 12 MHz frequency transducer. Regional and global contraction were evaluated in real time in the longitudinal and parasternal left ventricle (LV) sections. The cardiac systolic (LVSD) and diastolic (LVDD) dimensions were assessed using the M-mode. The left ventricular systolic function was evaluated by ejection fraction (EF) analysis (Armstrong and Ryan, 2011). The animals underwent echocardiography at the beginning and at the 1st and 10th month after infection, after being anesthetized with a combination of ketamine (50 mg/kg) and xylazine (10 mg/kg) intraperitoneally. The test was performed according to its standardization in small rodents, and the echocardiograms of healthy animals in the control group were considered normal (Salemi et al., 2005). A blinded examiner performed the echocardiography.

Quantitative real-time PCR analysis for inflammation, oxidative stress, and apoptosis: For gene expression analysis, we used the apex from the heart. Total RNA was isolated using miRNeasy Mini Kit (Qiagen, USA) according to manufacturer’s instructions. The quality of the samples was assessed by agarose gel electrophoresis and the concentration determined by reading it on a Qubit spectrophotometer (Thermo Fisher Scientific Inc, USA). The first strand of cDNA was synthesized from 1000 ng of total RNA using the Superscript II Reverse Transcriptase (Invitrogen by Thermo Fisher Scientific Inc, USA) and stored at -80°C. The gene expression levels of inflammatory cytokines (IL10, IFN-γ, and TNF), oxidative stress (NOX1, MnSOD, and iNOS), and apoptosis (BCL2 and CASP3) were determined by quantitative real-time PCR (qRT-PCR) using the TaqMan detection system. Reactions were performed in duplicate and run on a StepOnePlus RealTime PCR System (Applied Biosystems by Life Technologies, USA). The 2−ΔCt method was applied to calculate mRNA expression levels (Livak and Schmittgen, 2001).

Protein Expression Analysis by ELISA: We used commercial kits to evaluate protein quantification of inflammation (IL10, IFN-γ, and TNF) from MyBioSource (San Diego, California, USA) following guidance from the manufacturers. The protein determination was performed with 30 mg extract of cardiac tissue. The ELISA was performed in 96-well plates with standard sample (standard curve), target sample, and white sample (negative control) in duplicate. The reading was performed at 450 nm optical density (OD).

Detection of apoptosis by TUNEL: To detect apoptotic cells, we used *in situ* cell death detection, POD kit (Roche Applied Science) following guidance from the manufacturers. The assay was performed in paraffin sections (5 µm thick) from the medial slice of the hearts. Sections were pretreated with proteinase K (2.5 μg/mL) and endogenous peroxidase blockade was performed, followed by washing in running water. It was rinsed in phosphate buffered saline (PBS) and incubated in a humid atmosphere with TUNEL (deoxynucleotidyl transferase mediated dUTP nick end labeling assay) reaction. Then the slides were incubated with POD, washed in PBS, and stained with DAB. Staining was done with hematoxylin. All slides were scanned and quantified with a digital image analysis system, Scanscope (Aperio Technologies. California, USA).

Reactive Oxygen Species (ROS) Analysis: The assay was performed as previously described (Guido et al., 2017). The tissue was fixed, and the slides were deparaffinized and incubated with dihydroethidium reagent (5 mM) in a humidified darkroom. The slides were washed and placed to dry in the dark. After that, ProLong^®^ reagent was pipetted and the slides were kept in the dark to dry, and stored in a -20°C freezer, and analyzed using a fluorescence microscope. ROS were quantified using photos in 20x magnification through IrfanView software.

Statistical analysis: The Kolmogorov-Smirnov test was used for normality testing. For those variables with normal distribution, we performed two-tailed one-way analysis of variance (ANOVA) for the four group comparisons and *post-hoc* Tukey test for multiple comparisons. These data are presented as mean ± standard deviation. For those variables non-normally distributed, we applied the Kruskal-Wallis and Dunn tests for the differences. In these cases, the results are presented as median and 25th to 75th percentiles (Siegl and Castellan, 1988). The level of significance was 5%.

## Results

### Collagen Morphometry

**Figure 1:**
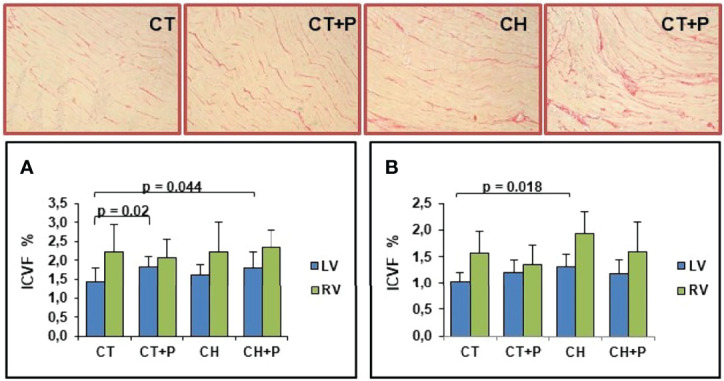
Photomicrograph of the myocardium stained with picrosirius red (in red the collagen marking), and graphic of Interstitial Collagen Volume Fraction (ICVF). **(A)** Collagen deposition in the acute phase (n=51); **(B)** collagen deposition in the chronic phase (n=40). CT - control group, CT+P – control group exposed to pollution, CH – chagasic group, CH+P – chagasic group exposed to pollution.

In the acute and chronic phase, we observed a greater deposition of collagen in the LV and RV of the chagasic groups compared with the control. We point out that in the acute phase, pollution increased fibrosis in the control group exposed to pollution compared with the control (p = 0.02). No increase in myocardial fibrosis was caused by pollution among Chagas groups (**Figure 1**).

### Echocardiography

**Figure 2:**
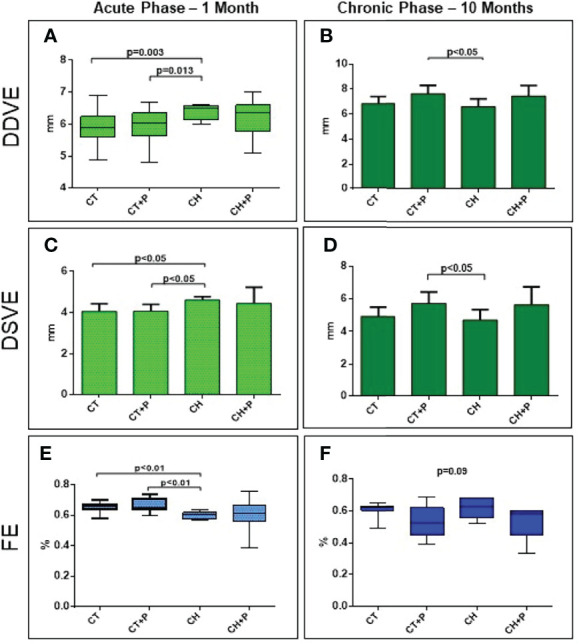
Left Ventricular diastolic diameter (LVDD), left ventricular systolic diameter (LVSD) and left ventricular ejection fraction (EF). **(A)** LVDD in the acute phase (n=62); **(B)** LVDD in the chronic phase (n=33). **(C)** LVSD in the acute phase (n=62); **(D)** LVSD in the chronic phase (n=33), **(E)** EF in the acute phase (n=62); **(F)** EF in the chronic phase (n=33). CT - control group, CT+P – control group exposed to pollution, CH – chagasic group, CH+P – chagasic group exposed to pollution.

The analysis of the heart geometry showed a difference in diastolic and systolic LV diameters at the chronic phase in CT+P compared with the CH group (p < 0.05). In the acute phase, there was an LV dilation in the CH group compared with controls (LVDD CH x CT, p = 0.003 and CH x CT+P, p = 0.013; LVSD CH x CT, p < 0.05 and CH x CT+P, p < 0.05). We did not observe any enlargement modulated by pollution. The ejection fraction (EF) did not worsen in the chronic phase (p = 0.09). In the acute phase, there was a decrease EF in animals in the CH groups (CH x CT, p < 0.01 and CH x CT+P, p < 0.01). There was no decrease in systolic function related to air pollution (**Figure 2**).

### Quantitative Real-Time PCR Analysis

#### Inflammation

**Figure 3:**
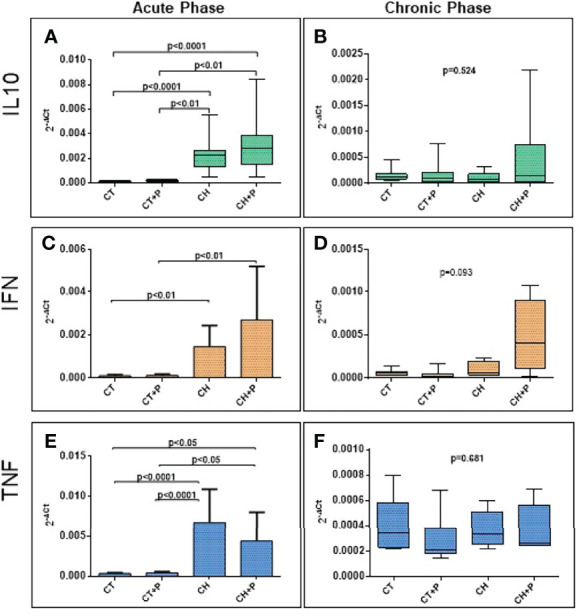
Absolute gene expression of inflammatory cytokines. **(A)** IL10 gene expression in the acute phase (n=49); **(B)** IL10 gene expression in the chronic phase (n=38). **(C)** IFN gene expression in the acute phase (n=49); **(D)** IFN gene expression in the chronic phase (n=38), **(E)** TNF gene expression in the acute phase (n=49); **(F)** TNF in the chronic phase (n=38). CT - control group, CT+P – control group exposed to pollution, CH – chagasic group, CH+P – chagasic group exposed to pollution.

The evaluation of IL10, IFN-γ, and TNF gene expression showed that in the acute phase, there was greater expression in the CH and CH+P groups compared with the control (p < 0.0001, for all genes, respectively). In the chronic phase, we did not find a statistical significance in the comparison between groups. There was no amplification of the expression of inflammatory cytokines by the pollution in either the acute or chronic phase (**Figure 3**).

#### Oxidative Stress

**Figure 4:**
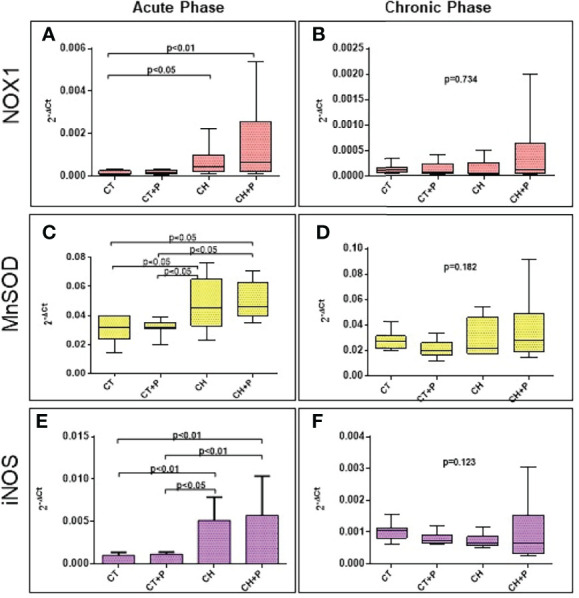
Gene expression of oxidative stress genes. **(A)** NOX1 gene expression in the acute phase (n=49); **(B)** NOX1 gene expression in the chronic phase (n=38). **(C)** MnSOD gene expression in the acute phase (n=49); **(D)** MnSOD gene expression in the chronic phase (n=38), **(E)** iNOS gene expression in the acute phase (n=49); **(F)** iNOS in the chronic phase (n=38). CT - control group, CT+P – control group exposed to pollution, CH – chagasic group, CH+P – chagasic group exposed to pollution.

In the acute phase, NOX1 expression was greater in chagasic groups compared with CT (CH x CT, p < 0.05 and CH+P x CT, p < 0.01). The expression of the MnSOD and iNOS genes also showed greater expression in the chagasic groups compared with the controls (p < 0.002). The chronic phase of the 3 genes was not statistically significant among the groups. Again, pollution did not increase the expression, in either the acute or chronic phase (**Figure 4**).

#### Apoptosis

**Figure 5:**
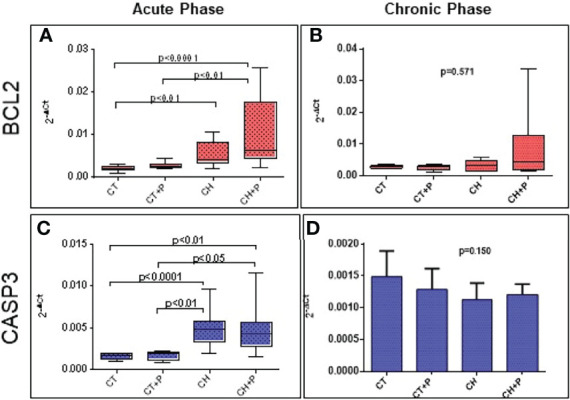
Gene expression of apoptosis genes. **(A)** BCL2 gene expression in the acute phase (n=49); **(B)** BCL2 gene expression in the chronic phase (n=38). **(C)** CASP3 gene expression in the acute phase (n=49); **(D)** CASP3 gene expression in the chronic phase (n=38). CT - control group, CT+P – control group exposed to pollution, CH – chagasic group, CH+P – chagasic group exposed to pollution.

The analysis of BCl2 and CASP3 gene expression shows an increase in chagasic groups compared with controls in the acute phase. There was a marked expression in the CH+P group compared with CH in the analysis of the acute phase of BCl2, but without statistical significance and with a marginal p of 0.054. The chronic phase did not show any difference in the comparison between the groups (**Figure 5**).

### Protein Expression – ELISA

#### Inflammation

**Figure 6:**
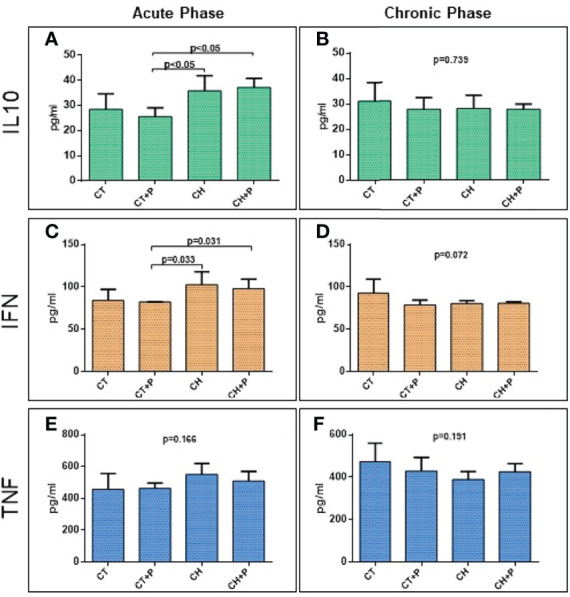
Protein expression of inflammatory cytokines. **(A)** IL10 protein expression in the acute phase (n=5); **(B)** IL10 protein expression in the chronic phase (n=5). **(C)** IFN protein expression in the acute phase (n=5); **(D)** IFN protein expression in the chronic phase (n=5), **(E)** TNF protein expression in the acute phase (n=5); **(F)** TNF protein expression (n=5). in the chronic phase. CT - control group, CT+P – control group exposed to pollution, CH – chagasic group, CH+P – chagasic group exposed to pollution.

Protein evaluation of cytokines IL10, IFN-γ, and TNF did not show increased expression in the chronic phase (IL10: p = 0.74; IFN-γ: p = 0.07; TNF p = 0.19). The acute phase analysis showed a significant increase in IL10 (CT+P x CH, p < 0.05 and CT+P x CH+P, p < 0.05) and IFN-γ (CT+P x CH, p < 0.03 and CT+P x CH+P, p < 0.03) (**Figure 6**). Pollution did not modulate the production of these cytokines.

### Apoptosis – TUNEL

**Figure 7:**
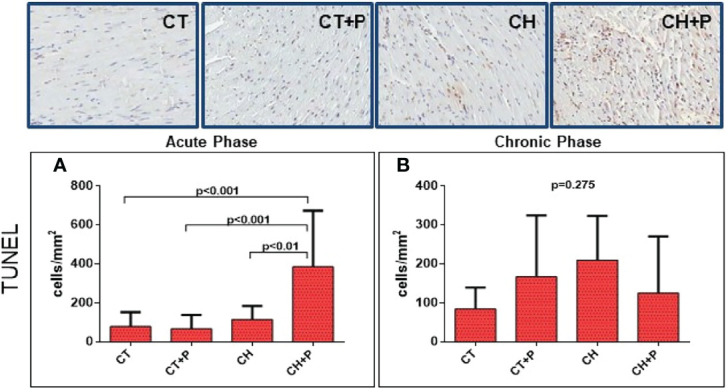
Photomicrograph with apoptotic nuclear in brown color of the myocardium for detection of DNA fragmentation using the TUNEL technique, and graph of the quantification of apoptosis. **(A)** Apoptosis (TUNEL) in the acute phase (n=51); **(B)** Apoptosis (TUNEL) in the chronic phase (n=40). CT - control group, CT+P – control group exposed to pollution, CH – chagasic group, CH+P – chagasic group exposed to pollution.

The evaluation of apoptosis by TUNEL we observed, in the acute phase, 2 times more apoptotic cells in CH+P than in CH (p < 0.01) showing possible pollution modulation in apoptosis. The chronic phase was not significant (p = 0.27) (**Figure 7**).

### ROS Analysis

**Figure 8:**
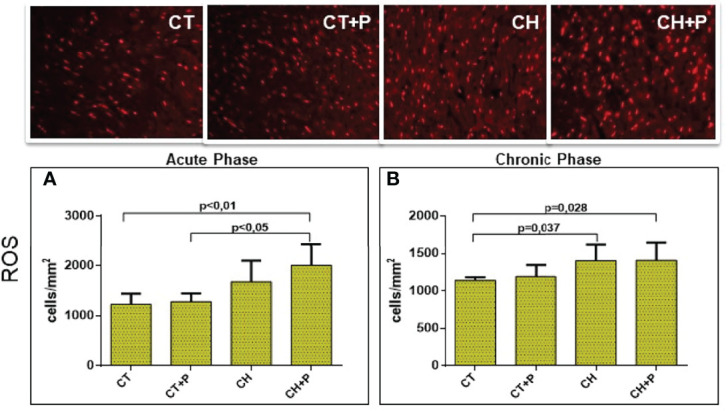
Photomicrograph of the myocardium for detection of reactive oxygen species (ROS) and graph of ROS quantification. **(A)** ROS in the acute phase (n=5); **(B)** ROS in the chronic phase (n=5). CT - control group, CT+P – control group exposed to pollution, CH – chagasic group, CH+P – chagasic group exposed to pollution.

The evaluation of the production of reactive oxygen species (ROS) showed an increase in chagasic groups in the acute phase (CT x CH+P, p < 0.01 and CT+P x CH+P, p < 0.05). In the chronic phase, we observed a higher production of ROS in the chagasic groups compared with the control (CT x CH, p = 0.037 and CT x CH+P, p < 0.028) (**Figure 8**).

### Survival

**Figure 9:**
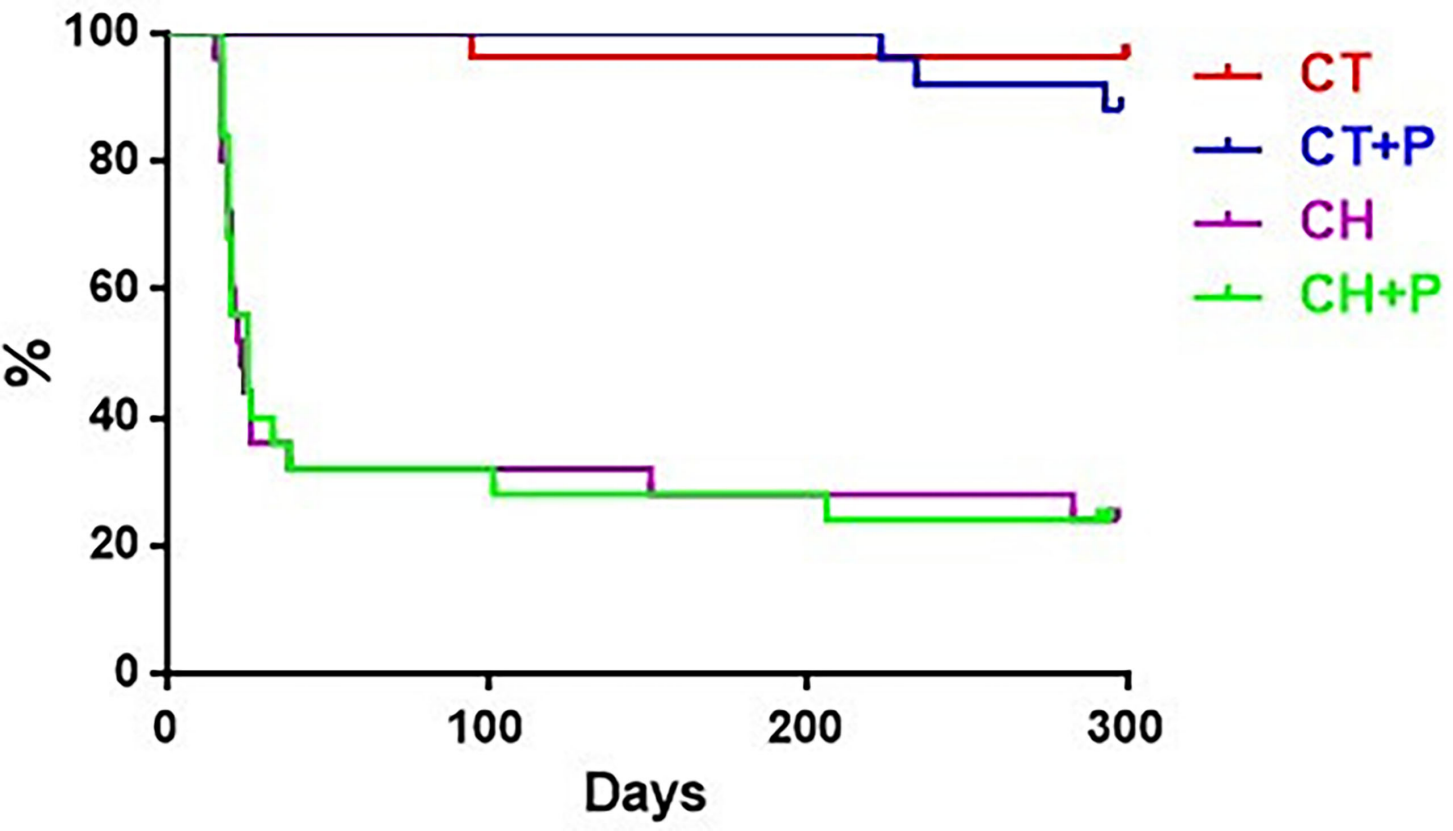
Graph of animal survival at 10 months compared to study groups. CT - control group (n=25), CT+P – control group exposed to pollution (n=25), CH – chagasic group (n=25), CH+P – chagasic group exposed to pollution (n=25).

The survival curve demonstrates high mortality - 68% of infected animals (Chagas and Chagas exposed to pollution) in the acute phase and 25% in the chronic phase. Control groups (control and control exposed to pollution) had only 4 deaths, remaining healthy until the end of the experiment. Air pollution did not change the survival of infected and uninfected animals (**Figure 9**).

## Discussion

Air pollution has been the subject of an increasing number of studies, due to its potential to cause mainly heart and lung diseases (Davoodabadi et al., 2019; Chen et al., 2019). On the other hand, Chagas disease has also been widely studied due to its high prevalence in Latin America and non-endemic regions, such as North America and Europe (Lidani et al., 2019).

The physiological mechanism that explains the damage caused by pollution in the heart is associated with the inhalation of particulate matter by the respiratory system, promoting local inflammation, which is transmitted to the circulation. Likewise, Chagas disease activates the inflammatory process, stimulated by T. cruzi and associated with oxidative stress, promotes cell death, and causes intense myocardial fibrosis. In our study, in the samples of the acute phase, greater gene expression was observed of the IL10, IFN-γ, and TNF genes in the chagasic groups compared with the controls. The analysis of the chronic phase did not show statistical significance when comparing the groups. It was the same for cytokine protein production with higher levels in chagasic groups, especially in the acute phase, where the parasitic burden is intense and the stimulus for inflammation is greater. In this acute phase, innate immunity is triggered by the parasite, promoting the exacerbated production of inflammatory cytokines, including IL12, TNF, and IFN-γ, as evidenced by Cunha Neto (Cunha-Neto et al., 1998) and Teixeira (Teixeira et al., 2002). The high production of IL-10, evidenced in our work, is of great importance in the acute phase for the control of parasitism and modulation of pro-inflammatory cytokines. Holscher et al. (Hölscher et al., 2000) demonstrated the importance of IL10 in a study with mice that did not produce this cytokine (Knockout or IL10 -/-) and infected with T. cruzi. IL-10 -/- homozygous mice showed greater lethality, from the third week post-infection, whereas the wild controls of this cytokine (IL-10 +/-) survived the acute infection. This, possibly, occurred precisely due to the suppression of the inhibition of inflammatory and parasitemia-inhibiting cytokines. In the chronic phase of Chagas disease, elevated levels of TNF and IFN-γ are described in chagasic patients, as a probable response to the persistence of the parasite due to the immune response (Cunha-Neto et al., 2005). In our study, although we found higher levels of these cytokines in the chronic phase, there was no significant difference between groups. This was possibly due to studying this phase at 10 months, which is a long time in the life of the Syrian hamster resulting in inflammatory cytokines falling. Another possible cause would be the high mortality of our study in the acute phase, with only the healthiest animals surviving because they probably had less aggressive infection. In Chagas disease, we have not found an exacerbation of the production of these cytokines by pollution. In the acute phase, there was an increase in inflammatory cytokines in Chagas groups, but pollution did not amplify this response. In the chronic phase, the groups had homogeneous expression of cytokines, and pollution also did not promote an increase in this gene expression. This non-amplification, due to pollution, may be related to a very intense antigenic stimulus of infection by T. cruzi and the immune response caused by it, and pollution in this scenario has added little to amplify this response.

In relation to protein analyzes of inflammatory cytokines studied in cardiac tissue, the protein expression of IL10 and IFNγ is increased in the chagasic groups, in the acute phase of the disease, compared with the control groups. In the chronic phase, there was no change in this expression in any of the analyzed cytokines. TNF showed an increase of 20% in Chagas groups in relation to controls, but without a statistical difference. Mendonça (Mendonça et al., 2019) studying the same inflammatory cytokines in cardiac tissue of Wistar rats also found a significant increase in IL-10, TNF, and IFNγ in the groups infected in the acute phase of the disease. The higher protein expression of IL-10 and IFN-γ, in the acute phase, suggests a compensatory stimulus for these two cytokines, since IL-10 regulates the inflammatory response that is exacerbated in this phase. Oliveira-Fonoff (de Oliveira-Fonoff et al., 2017) showed an increase in the inflammatory infiltrate in the heart of healthy rats exposed to pollution. In our study, pollution did not increase the levels of cytokines in the hearts of infected or noninfected animals. Once again, the stimulation of inflammation by T. cruzi may be so intense that pollution has not added further activation.

The interrelation of inflammatory pathways and oxidative stress occurs through the interaction of mediators and signalers. Cellular damage caused by ROS is well described in the literature, and several authors have already demonstrated the increase in oxidative stress with exposure to air pollution (Kim et al., 2016; Bourdrel et al., 2017). Other studies have shown the same response in the scenario of infection by T. cruzi (Paiva et al., 2018; Novaes et al., 2018). We evaluated the role of Nox-1 (superoxide builder) and observed greater expression in Chagas groups compared with controls, but without pollution interference. Similar results were presented by Gray (Gray et al., 2016) who demonstrated, in another inflammatory scenario, in the case of atherosclerosis, an increase in the expression of Nox-1 described in patient plates that had cardiovascular events or diabetes mellitus. Such behavior of the oxidative stress dynamics in Chagas disease has not been previously shown in the literature. Again, Oliveira-Fonoff (de Oliveira-Fonoff et al., 2017) demonstrated an increase in glutathione in healthy animals exposed to pollution. In our Chagas model, the pollution exposed infected animals did not have greater activation of oxidative stress in the studied variables, possibly due to the intense stimulus caused by T. cruzi infection.

Regarding oxidative stress, some evidence points out that antioxidant enzymes are essential in regulating inflammation, and manganese superoxide dismutase (MnSOD) acts in this way. Wen et al. (Wen and Garg, 2018), in order to assess the effects of MnSOD deficiency in oxidative damage in chronic Chagas disease, used wild mice and MnSOD (MnSOD +/-) deficient mice infected with T. cruzi and demonstrated that this deficiency decreases myocardial expression and MnSOD activity and significantly increases oxidative damage in mitochondria. Our work showed a higher production of MnSOD in chagasic groups, in the acute phase, compared with controls. This increase, possibly, occurred in response to the increase in ROS, as we observed in relation to Nox-1 and ROS analysis. Although the gene expression of MnSOD was greater in Chagas groups, pollution was once again unable to exacerbate this expression.

The evaluation of the ROS in cardiac tissue fixed in formaldehyde and incubated with DHE (dihydroetide). Our findings showed a higher production of ROS in the infected groups, confirming the stimulus to oxidative stress. Both in in the acute and chronic phase, we observed greater production of ROS in the chagasic groups compared with the control groups. The same result can be seen in the work of Novaes (Novaes et al., 2017) who, using a Wistar rat model in the acute phase of Chagas disease, observed an increase in ROS in the animals of the infected groups. In our study, we noticed that the chagasic group exposed to pollution showed a higher production of ROS, despite not showing a statistical difference, between the infected groups. Probably, the oxidative damage caused by Chagas disease in the studied phases is so intense that, although pollution has increased the production of ROS in the Chagas group, it was not decisive to significantly amplify this pathway.

Nitric oxide (NO) plays a central role in several pathophysiological processes. Induced nitric oxide synthase (iNOS) is a NOS induced by cytokines and lipopolysaccharides, in the endothelium and vascular smooth muscle, and acts on parasites and tumor cells. In our study, we observed a high expression of iNOS in chagasic groups compared with controls in the acute phase. In the chronic phase, there was no statistical difference in the comparison between groups. Vasconcelos (Vasconcelos et al., 2017) evaluated the administration of dimethylsphingosine (DMS - mediator of cellular events during the inflammatory response) in mice chronically infected with T. cruzi and also demonstrated that there was a greater expression of iNOS in the heart of infected and solution-treated mice saline compared with the uninfected group. However, once again, this high production of iNOS in infected animals was not aggravated by pollution.

The analysis of apoptosis included the evaluation of the gene expression of the BCL2 and CASP3 genes, in addition to the TUNEL technique. Our results, referring to the expression of BCL2 (anti-apoptotic), showed an increase of this expression in the chagasic groups in comparison to the controls, in the acute phase of the experiment. The pollution did not intensify the expression of this gene, although the p among chagasic groups was borderline (p = 0.05), suggesting that, as in the production of ROS, which was higher in the infected group exposed to pollution, but without statistical significance, the stimulus to apoptosis by pollution in the infected groups seems to be present. The same pattern can be observed in the analysis of gene expression of caspase 3 (pro-apoptotic), with greater expression in chagasic groups compared with controls, in the acute phase, without intensification by pollution. Seriani (Seriani et al., 2016) showed an inversely proportional expression of these 2 genes, where there was a predominance of caspase 3 expression compared with BCL2. Seriani used bronchial epithelium cells exposed to diesel pollution and noted that apoptotic factors are superimposed on non-apoptotic factors. It seems that there is a competition between anti-apoptotic and pro-apoptotic factors, acting intensely in these animals studied, with a slight predominance of anti-apoptotic factors, most likely, induced by inflammatory cytokines. In the chronic phase, both genes did not promote a significant difference in the expression of the studied groups.

The TUNEL technique also suggests an apoptotic process in the acute phase of the disease, when we observe a greater number of apoptotic cells in the chagasic group exposed to pollution compared with the other groups. With this technique, we were able to observe the influence of pollution on the apoptosis of cardiac cells. Carbajosa (Carbajosa et al., 2017), using slices of thymus from mice infected with T. cruzi, on different days post-infection, observed a significant increase in apoptotic thymocytes, at 21 days post-infection, that is, a high apoptosis in the acute phase.

Inflammation, oxidative stress, and apoptosis pathways are possible candidates that promote exacerbated collagen deposition in the myocardium, leading to structural remodeling and acting as a mechanism of cardiac dysfunction (Azevedo et al., 2016). Exposure to particulate material activates the same pathways and, in addition to causing ventricular remodeling and worsening cardiac fibrosis (Wold et al., 2012), it also causes loss of the heart’s contractility. In our study, analyzing interstitial collagen, we observed marked collagen deposition in chagasic groups compared with controls, both in the right and left ventricles in acute and chronic phases. We observed an increase in fibrosis comparing the groups CT and CT+P in the LV. This finding suggests that, in fact, pollution stimulates the accumulation of collagen in the myocardium. Wold (Wold et al., 2012), using mice exposed to pollution (PM_2.5_), showed the impact of collagen deposition in the heart, where he observed a 166% increase in collagen deposition in mice exposed to MP_2.5_, compared with mice subjected to inhalation of filtered air. Our results showed that there is interference from pollution between the control groups, but we did not observe the same results when comparing the chagasic groups with each other.

Echocardiographic analysis provides data on geometry, structure, and cardiac function. Our geometry results showed a dilation of the LV (DDVE and DSVE) in both chagasic groups in the acute phase of the disease. In the chronic phase, we did not observe this dilation, possibly related to the fact that at 10 months the surviving animals are the healthiest. In both phases, pollution did not increase the dilation among the infected groups. Oliveira-Fonoff (de Oliveira-Fonoff et al., 2017) compared the LV geometry between groups of infarcted animals and those exposed to pollution and also did not observe any dilation due to pollution between the groups. Regarding systolic function (EF), we observed a decrease in this function in the chagasic groups, during the acute phase, as expected. Although we have shown a significant decrease in this function, in the acute phase, between chagasic groups and control groups, pollution did not interfere with the loss of ventricular function. In the chronic phase, the loss of function is not statistically significant when comparing the groups. Again, possibly due to the high mortality in the acute phase, the animals surviving at 10 months were the healthiest.

## Conclusions

Air pollution did not increase collagen deposition, did not worsen ventricular function or dilation, did not show changes in inflammation pathways or oxidative stress in this model of Chagas cardiomyopathy. There was possible modulation of the apoptotic pathway by pollution both in gene expression and in the counting of apoptotic cells, which leads us to believe that pollution affects the aforementioned pathway, but with no strong stimulus as needed to amplify the cell loss and reparative fibrosis.

## Data Availability Statement

The original contributions presented in the study are included in the article/supplementary files. Further inquiries can be directed to the corresponding author.

## Ethics Statement

This study was reviewed and approved by Comissão de Ética no Uso de Animais (CEUA).

## Author Contributions

KF – conceptualization, data curation, investigation, methodology, performed the statistical analysis, wrote the first draft of the manuscript, read, and approved the submitted version. FP - data curation, investigation, methodology, performed the statistical analysis, read, and approved the submitted version. OR - data curation, investigation, methodology, read and approved the submitted version. VH - investigation, methodology, read and approved the submitted version. BI - contributed to conception and design of the study, read, and approved the submitted version. FF - contributed to conception and design of the study, read, and approved the submitted version. DR - contributed to conception and design of the study, read, and approved the submitted version. PS - contributed to conception and design of the study, read, and approved the submitted version. CM - contributed to conception and design of the study, read, and approved the submitted version. FR - conceptualization, data curation, organized the database, wrote the first draft of the manuscript, funding acquisition, read and approved the submitted version. All co-authors participated in the formulation of the manuscript or critically reviewed contributing to its intellectual content, approved the version to be published and agreed to be responsible for all aspects of the work, ensuring that issues related to the accuracy or integrity of any part of the work were properly investigated and resolved.

## Funding

This work was supported by Fapesp (Fundacao de Amparo à Pesquisa) n° 2014/23941-8

## Conflict of Interest

The authors declare that the research was conducted in the absence of any commercial or financial relationships that could be construed as a potential conflict of interest.

## Publisher’s Note

All claims expressed in this article are solely those of the authors and do not necessarily represent those of their affiliated organizations, or those of the publisher, the editors and the reviewers. Any product that may be evaluated in this article, or claim that may be made by its manufacturer, is not guaranteed or endorsed by the publisher.
